# Silencing of secreted phosphoprotein 1 attenuates sciatic nerve injury‐induced neuropathic pain: Regulating extracellular signal‐regulated kinase and neuroinflammatory signaling pathways

**DOI:** 10.1002/iid3.1132

**Published:** 2024-02-02

**Authors:** Haiyu Xie, Feng Lu, Xiaoling Li, Enfu Wang, Jiao Mo, Weidong Liang

**Affiliations:** ^1^ Department of Anesthesiology The First Affiliated Hospital of Gannan Medical University Ganzhou City Jiangxi Province China

**Keywords:** ERK pathway, neuropathic pain, sciatic nerve injury, SPP1

## Abstract

**Background:**

Neuropathic pain (NP) is a chronic pathological pain that affects the quality of life and is a huge medical burden for affected patients. In this study, we aimed to explore the effects of secreted phosphoprotein 1 (SPP1) on NP.

**Methods:**

We established a chronic constriction injury (CCI) rat model, knocked down SPP1 via an intrathecal injection, and/or activated the extracellular signal‐regulated kinase (ERK) pathway with insulin‐like growth factor 1 (IGF‐1) treatment. Pain behaviors, including paw withdrawal threshold (PWT), paw withdrawal latency (PWL), lifting number, and frequency, were assessed. After sacrificing rats, the L4‐L5 dorsal root ganglion was collected. Then, SPP1 levels were determined using quantitative polymerase chain reaction (qPCR) and western blot analysis. The levels of interleukin (IL)‐1β, tumor necrosis factor (TNF)‐α, IL‐6, IL‐10, epidermal growth factor (EGF), vascular endothelial growth factor (VEGF), and transforming growth factor (TGF)‐β were determined using qPCR and enzyme‐linked immunosorbent assay. The levels of ERK pathway factors were determined via western blot analysis.

**Results:**

We found that CCI decreased PWT and PWL, increased the lifting number and frequency, and upregulated SPP1 levels. The loss of SPP1 reversed these CCI‐induced effects. Additionally, CCI upregulated IL‐1β, TNF‐α, IL‐6, EGF, and VEGF levels, downregulated TGF‐β levels, and activated the ERK pathway, while silencing of SPP1 abrogated these CCI‐induced effects. Moreover, IGF‐1 treatment reversed the effects of SPP1 loss.

**Conclusions:**

The data indicate that silencing SPP1 attenuates NP via inactivation of the ERK pathway, suggesting that SPP1 may be a promising target for NP treatment.

## Highlights

- Depletion of secreted phosphoprotein 1 (SPP1) alleviates inflammation response.
- Knockdown SPP1 inactivated the extracellular signal‐regulated kinase (ERK) pathway.
- Silencing of SPP1 relieves neuropathic pain by regulating the ERK pathway.

## Graphical abstract

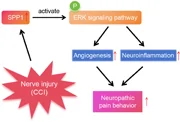

## 1 INTRODUCTION

Neuropathic pain (NP) is an epidemic of chronic pain that affects approximately 10% of global population.^1^ NP is characterized by hyperalgesia, allodynia, and spontaneous pain.^2^ Spontaneous pain is persistent or intermittent and may be accompanied by evoked pain.^3^ In addition to nerve injury, age, sex, and emotion are risk factors for the occurrence of NP, which usually develops in the nerve roots, plexuses, and nerve trunks.^4^, ^5^ To date, the diagnosis of NP has not been standardized, and it is easily confused with non‐NP.^6^ Current treatment strategies are not fully effective in treating NP, and drug inefficiency and resistance further increase the difficulty of NP management.^3^, ^7^ Thus, there is an urgent need to clarify the mechanism underlying NP progression to develop more effective treatment methods.

Secreted phosphoprotein 1 (SPP1; also known as OPN, BNSP, BSPI, and ETA‐1) is a glycoprotein with multiple functions. The protein encoded by *SPP1* is a type of osteopontin that is closely linked to the connection between osteoclasts and mineralized bone matrix.^8^ SPP1 levels are upregulated in osteoblasts. SPP1 levels are also highly upregulated during the pathogenesis and progression of muscular dystrophy in mice and humans.^9^ SPP1 is secreted into the extracellular matrix and mediates cell adhesion and migration.^10^ Due to cell adhesion, SPP1 expression is closely related to tumorigenesis and metastasis, suggesting that it can be used as a diagnostic and prognostic biomarker of cancer.^11^, ^12^, ^13^ Moreover, SPP1 is associated with multiple types of pain, such as diabetic NP, cancer pain, and low back pain.^14^, ^15^, ^16^ Importantly, a previous study has revealed that SPP1 expression is increased after sciatic nerve injury and regulating nerve degeneration and regeneration.^17^ However, the role of SPP1 in sciatic nerve injury‐induced NP and the underlying molecular mechanism has not been fully elucidated. Additionally, NP is associated with a pro‐inflammatory state, which involves the release of multiple pro‐inflammatory factors, such as tumor necrosis factor (TNF)‐α, interleukin (IL)‐1β, IL‐6, and IL‐17, and the inhibition of anti‐inflammatory factors, such as IL‐4, IL‐10, transforming growth factor (TGF)‐β.^18^ Angiogenesis is evident in inflammation of neurons. In spinal cord, central neuroinflammation and angiogenesis occurs in conjunction, which is related to vascular endothelial growth factor (VEGF) pathway.^19^ Therefore, we focused on the regulation of inflammatory factors and angiogenesis‐related factors by SPP1.

The MAP kinse‐ERK kinase (MEK)/extracellular signal‐regulated kinase (ERK) cascade is a well‐characterized mitogen‐activated protein kinase (MAPK) pathway involved in cell proliferation and survival.^20^ The ERK pathway is activated under various conditions, such as malignancy, inflammation, and fibrotic disease.^21^, ^22^, ^23^ After nerve injury, ERK phosphorylation levels are increased in neurons, microglia, astrocytes, and large dorsal root ganglia neurons. ERK signaling pathway is a promising therapeutic target for NP treatment, and inhibiting ERK synthesis can relieve pain.^24^ Increasing evidence has reported that SPP1 can regulate the ERK pathway in the pathological process of disease.^17^, ^25^ Therefore, we sought to investigate whether SPP1 affects the ERK pathway in NP.

Here, we aimed to explore the involvement of SPP1 in NP and the underlying mechanism. We hypothesized that SPP1 was involved in NP progression by regulating inflammation and angiogenesis, which may by be related to the ERK signaling pathway. Thus, this study may provide a therapeutic target for NP.

## 2 MATERIALS AND METHODS

### 2.1 Animals

Adult male Sprague‐Dawley rats (weight: 180–200 g) were kept under 12 h light/12 h dark cycle at 22°C and provided enough food and water. All rats were acclimated to the conditions for 7 days until operation. Then the rats were divided into six groups (six rats per group): (1) sham, rats operated without ligation; (2) chronic constriction injury (CCI) model, rats operated with bilateral sciatic nerve ligation; (3) CCI + lentivirus negative control (Lv‐sh‐nc), rats were intrathecal injected with Lv‐sh‐nc, and CCI model was established; (4) CCI + Lv‐sh‐SPP1 (lentivirus carrying short hairpin [Sh] RNA targeting SPP1), rats were intrathecal injected with Lv‐sh‐SPP1, and CCI model was established; (5) CCI + Lv‐sh‐SPP1 + normal saline (Nacl), rats were intrathecal injected with Lv‐SPP1, followed by intrathecal injection with normal saline, and CCI model was established; and (6) CCI + Lv‐sh‐SPP1 + insulin‐like growth factor 1 (IGF‐1) groups, rats were intrathecal injected with Lv‐SPP1, followed by intrathecal injection with IGF‐1, and CCI model was established. The schematic diagram of experimental design is shown in Supporting Information: Figure 1.

### 2.2 CCI operation

Bilateral sciatic nerve ligation was performed to establish a CCI rat model as previously described.^26^, ^27^ Briefly, the rats were anesthetized with 50 mg/kg sodium pentobarbital, and the sciatic nerves on both sides of the hind limbs were exposed and separated from the surrounding soft tissue. The sciatic nerves were ligated using 4‐0 catgut thread at four sites (1 mm interval). Ligation was performed by a single operator to ensure a similar ligation tightness. Rats with exposed sciatic nerves, without ligation, served as the sham group. After the surgery, the wound was closed. The rats were housed in single cages. After determining the pain behaviors, L4‐L5 dorsal root ganglion were obtained cooled in liquid nitrogen, and stored at −80°C.

### 2.3 Determination of pain behaviors

Pain behaviors were measured on the day of surgery and 3, 7, and 14 days after surgery as previously described.^28^ The paw withdrawal threshold (PWT) was determined by stimulating the rats with von Frey monofilaments. Rats were left alone in a quiet and transparent cage for 15–30 min. Electronic von Frey was used to stimulate the plantar surfaces of the left and right hind claws of rats. Each rat was stimulated thrice. When the rats showed foot contraction behavior, the acupuncture intensity (*g*) was recorded. Moreover, at each stimulus force, the withdrawal frequency was determined.

Paw withdrawal latency (PWL) and paw lifting number were measured using a heat radiometer to irradiate the surface of the hind limb sole. The time from the beginning of irradiation to foot contraction behavior was recorded. Set the maximum stop time to 20 s to avoid tissue damage. The number of paw lifts within 5 min was recorded. Each rat was checked thrice.

The frequency was used to evaluate the cold sensitivity of the rats. One drop of acetone was placed on each hind paw. The number of foot contractions was also recorded. Each experiment was repeated thrice, and each interval was of 3 min.

The study was double blind. The measurers and recorders did not know which group each rat belonged to, and also did not know grouping details.

### 2.4 Intrathecal injection

The fragments of ShRNA‐SPP1 (CCTAAGAGTAAGGAAGATGAT) and its negative control (sh‐nc: CAACAAGATGAAGAGCACCAA) were purchased from Genepharma. These sequences were ligated into pLKO.1‐puro vector. HEK293T cells were transfected with pLKO.1‐shRNA constructs, psPAX2 packing plasmid, and pMD2.G envelope plasmid for 72 h. The supernatant containing the packaged particles was collected and the lentivirus were concentrated using ultracentrifugation. Intrathecal injection was performed using a modified version as previously described.^29^ The rats were anesthetized by intraperitoneal injection of 1% pentobarbital sodium (40 mg/kg), and 10 μL Lv‐sh‐SPP1 and Lv‐sh‐nc were intrathecal injected before CCI model establishment. The rats in the sham and CCI groups were intrathecally injected with 10 μL sterile saline. To activate the ERK pathway, IGF‐1 (ERK pathway agonist) was injected into the rats after Lv‐sh‐SPP1 injection, while saline (NaCl) was used as the negative control. Intrathecal injection was conducted at the L4‐L5 intervertebral space.

### 2.5 Enzyme‐linked immunosorbent assay (ELISA)

Dorsal root ganglion samples were lysed on ice. The supernatant was collected via centrifugation at 10,000*g* for 15 min. The levels of IL‐1β (RLB00), TNF‐α (RTA00), IL‐6 (R6000B), IL‐10 (R1000), epidermal growth factor (EGF) (DY3214), VEGF (RRV00), and TGF‐β (MB200) were determined using their specific ELISA kits (R&D Systems).

### 2.6 Quantitative polymerase chain reaction (qPCR)

Total RNA was extracted using the TRIzol reagent (Invitrogen). The M‐MLV reverse transcriptase kit (Invitrogen) was used to performed the reverse transcription of RNA. qPCR was conducted using SYBR Green qPCR Master Mix (MedChemExpress). All procedures were performed according to the manufacturer's instructions. The relative expression of mRNA was normalized to that of glyceraldehyde‐3‐phosphate dehydrogenase and calculated using the 2^−ΔΔCt^ method. The sequences of primers are shown as follows: SPP1 F: 5′‐GAGCAAACAGACGATGTGGA‐3′ and R: 5′‐GAAATCGGTGACCAGCTCAT‐3′; IL‐1β F: 5′‐GAGTGTGGATCCCAAGCAAT‐3′ and R: 5′‐TACCAGTTGGGGAACTCTGC‐3′; TNF‐α F: 5′‐CAGCCGATGGGTTGTACCTT‐3′ and R: 5′‐GGCAGCCTTGTCCCTTGA‐3′; IL‐6 F: 5′‐CCACGGCCTTCCCTACTTC‐3′ and R: 5′‐TGGGAGTGGTATCCTCTGTGAA‐3′; IL‐10 F: 5′‐GATGCCCCAGGCAGAGAA‐3′ and R: 5′‐CACCCAGGGAATTCAAATGC‐3′; EGF F: 5′‐GAAACTGTTGGGAGAGGAATCG‐3′ and R: 5′‐ AGCAAGGCAAAGGCTTAGAG‐3′; VEGF F: 5′‐ ATTTCTGGGATTCCTGTAG‐3′ and R: 5′‐CAGTGAAGACACCAATAAC‐3′; TGF‐β F: 5′‐ GGACACGCAGTACAGCAAG‐3′ and R: 5′‐GAGCGCACGATCATGTTGG‐3′; and GAPDH F: 5′‐CGACAGTCAGCCGCATCTT‐3′and R: 5′‐CCAATACGACCAAATCCGTTG‐3′.

### 2.7 Western blot

Proteins were extracted using the ice‐cold radioimmunoprecipitation assay lysis buffer, and protein concentration was determined using a BCA kit (Applygen). Equal amounts of protein were added into each lane, separated by 10% sodium dodecyl sulfate‐polyacrylamide gel electrophoresis, and transferred onto polyvinylidene fluoride (PVDF) membranes. After blocking with 5% skim milk, the PVDF membranes were incubated with primary antibodies (Abcam) at 4°C overnight, followed by incubation with horseradish peroxidase‐conjugated secondary antibody (Abcam) at room temperature for 1 h. Protein bands were detected using ECL luminescence reagent (Sangon) and quantified using the ImageJ 1.8.0 software (National Institutes of Health).

The primary antibodies used here were SPP1 antibody (ab307994, 1:1000), GAPDH antibody (ab181602, 1:10000), serine/threonine kinase (AKT; ab8805, 1:500), phosphorylated (p)‐AKT (ab38449, 1:500), MAPK (MEK; ab109556, 1:1000), p‐MEK (ab96379, 1:1000), ERK (ab184699, 1:10000), p‐ERK (ab76299, 1:10000). The secondary antibody was goat anti‐rabbit (ab6721, 1:5000).

### 2.8 Statistical analysis

All data are shown as the mean ± standard deviation using GraphPad Prism 6 software. Significance was analyzed by one‐way analysis of variance (ANOVA) and Student's *t*‐test. *p* < .05 was considered to be significant difference.

## 3 RESULTS

### 3.1 SPP1 expression is upregulated in the CCI rat model

**Figure 1:**
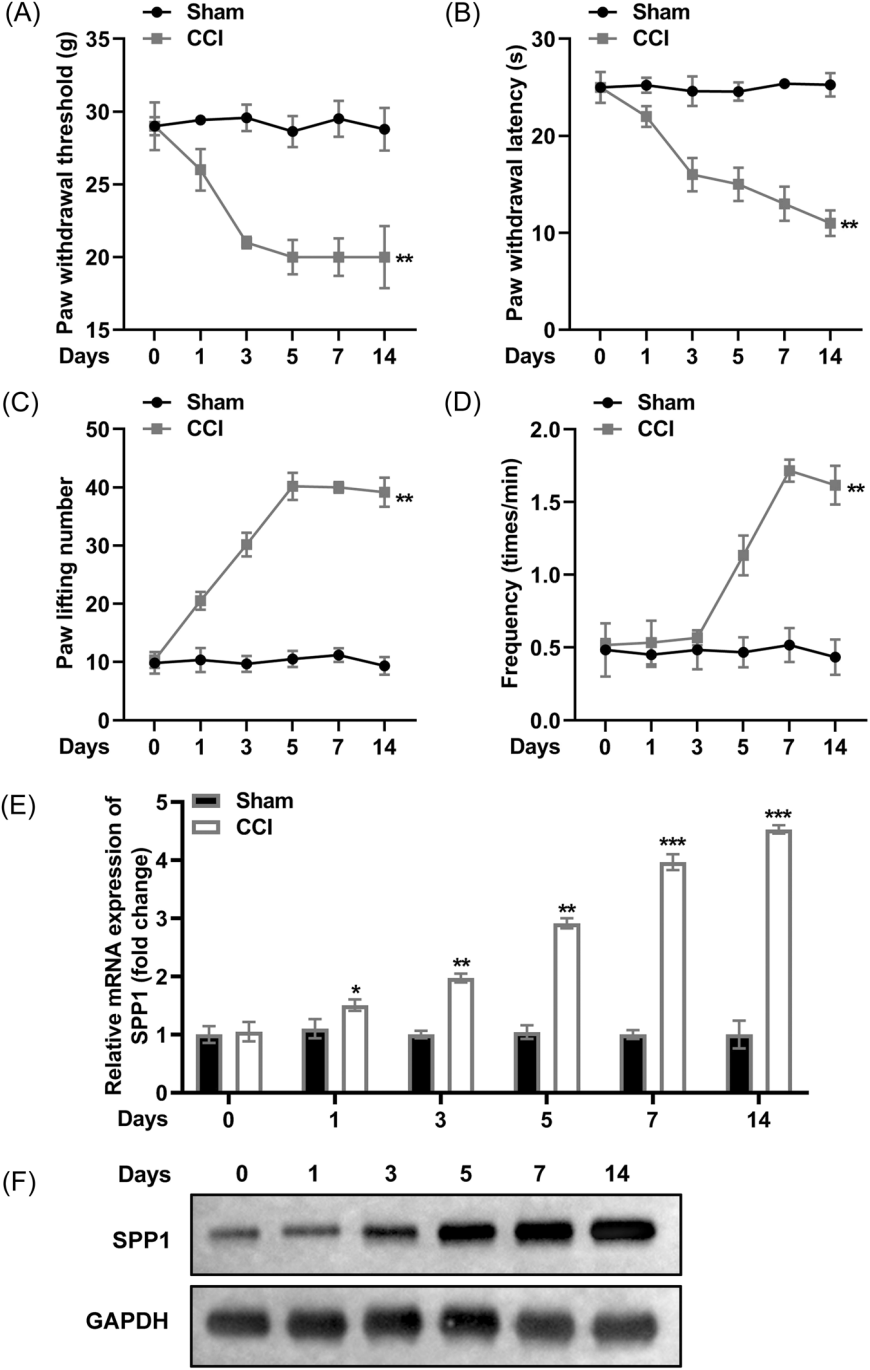
Secreted phosphoprotein 1 (SPP1) expression is upregulated in the chronic constriction injury (CCI) rat model. (A) Paw withdrawal threshold, (B) paw withdrawal latency, (C) paw lifting number, and (D) frequency were determined after CCI operation for 0, 1, 3, 5, 7, and 14 days. SPP1 levels were determined via (E) quantitative polymerase chain reaction and (F) western blot. *p < .05; **p < .01; ***p < .001.

Initially, the pain behaviors were measured. As shown in Figure 1A, CCI induced a significant decrease in PWT from Day 0 to Day 5, whereas PWT remained almost unchanged from Day 5 to Day 14. Similarly, PWL was markedly lower in the CCI group than in the sham group (Figure 1B). Furthermore, CCI significantly increased the number and frequency of paw lifts (Figure 1C,D). In addition, SPP1 levels were significantly elevated in the CCI group in a time‐dependent manner compared to those in the sham group (Figure 1E,F). The results demonstrated that CCI induces pain, and SPP1 expression was increased in CCI rats.

### 3.2 Knockdown SPP1 relieves NP in CCI‐induced rats

**Figure 2:**
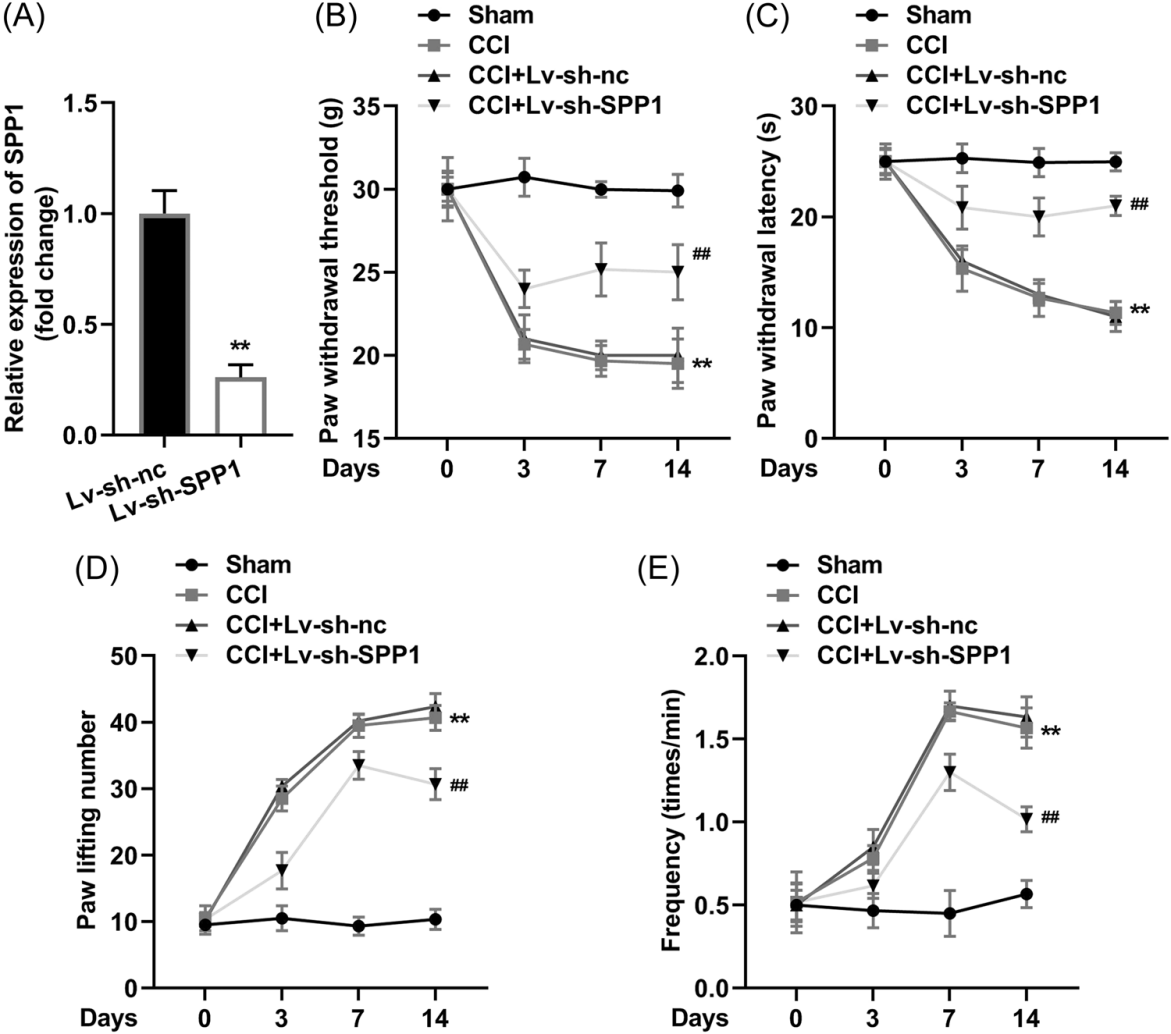
Knockdown secreted phosphoprotein 1 (SPP1) relieves neuropathic pain in chronic constriction injury (CCI)‐induced rats. (A) Levels were determined via quantitative polymerase chain reaction following transfection. (B) Paw withdrawal threshold, (C) paw withdrawal latency, (D) paw lifting number, and (E) frequency were measured after SPP1 knockdown. **p < .01; ^##^ *p* < .01.

SPP1 levels were downregulated following the transfection of Lv‐sh‐SPP1 compared to those in the Lv‐sh‐nc group (Figure 2A). Knockdown of SPP1 significantly increased the PWT and PWL in CCI rats (Figure 2B,C). In contrast, the number and frequency of paw lifts were markedly decreased in CCI‐induced rats due to the loss of SPP1 (Figure 2D,E). Taken together, interference with SPP1 can alleviate CCI‐induced pain.

### 3.3 Silencing of SPP1 inhibits the release of pro‐inflammatory factors in CCI‐induced rats

**Figure 3:**
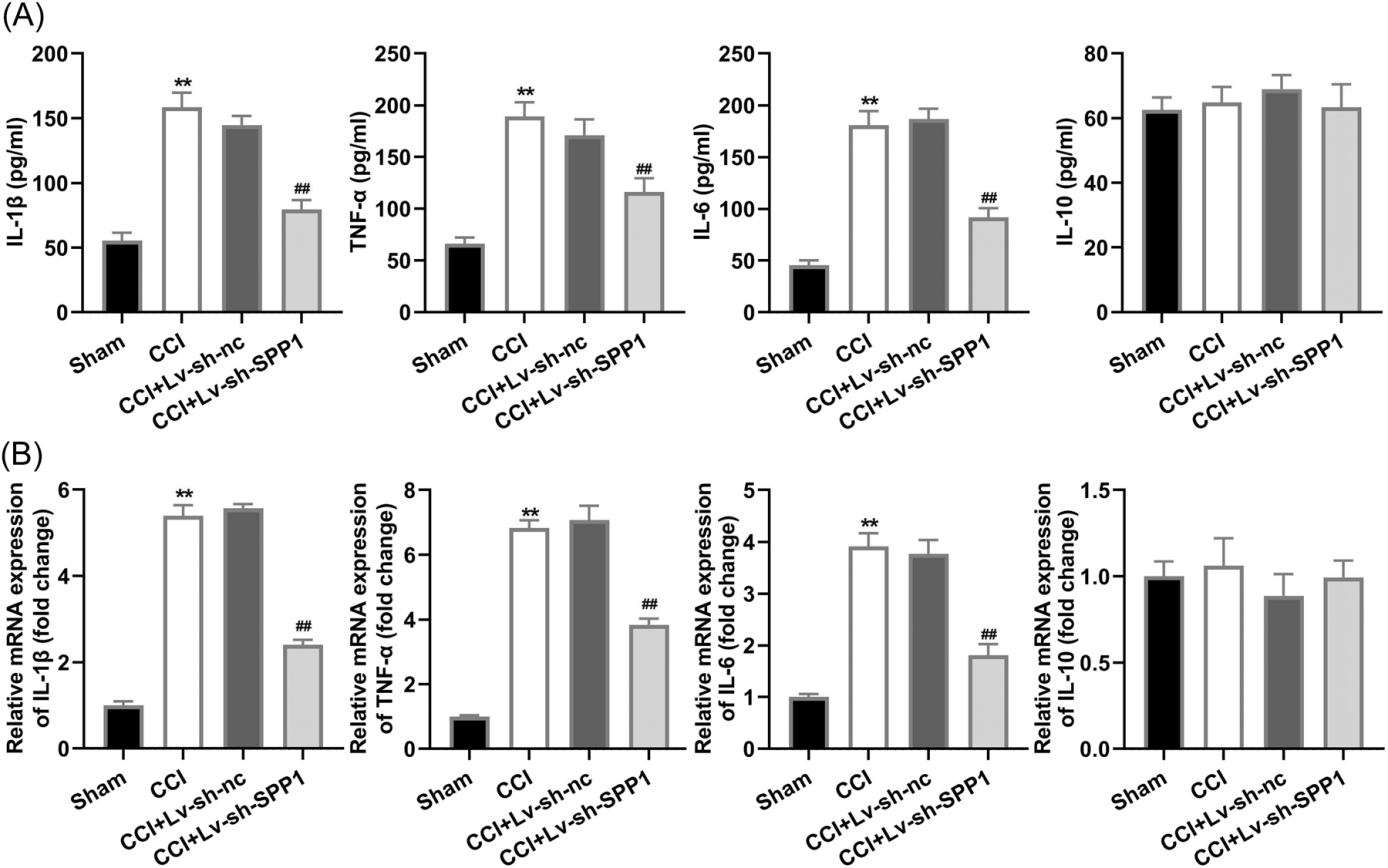
Depletion of secreted phosphoprotein 1 (SPP1) alleviates the inflammation response. The levels of inflammatory factors, interleukin (IL)‐1β, tumor necrosis factor (TNF)‐α, IL‐6, and IL‐10, were determined via (A) enzyme‐linked immunosorbent assay and (B) quantitative polymerase chain reaction. **p < .01; ^##^ *p* < .01.

NP is associated with a pro‐inflammatory state, therefore, the levels of inflammatory cytokines in the dorsal root ganglion were measured using qPCR and ELISA. The expression levels of IL‐1β, TNF‐α, and IL‐6 were significantly elevated by CCI, which were markedly abrogated by the knockdown of SPP1 (Figure 3A,B). IL‐10 levels were not affected by CCI or SPP1 in rats (Figure 3A,B), suggesting that SPP1 knockdown inhibits inflammation response in CCI rats.

### 3.4 Silenced SPP1 inhibits angiogenesis in CCI‐induced rats

**Figure 4:**
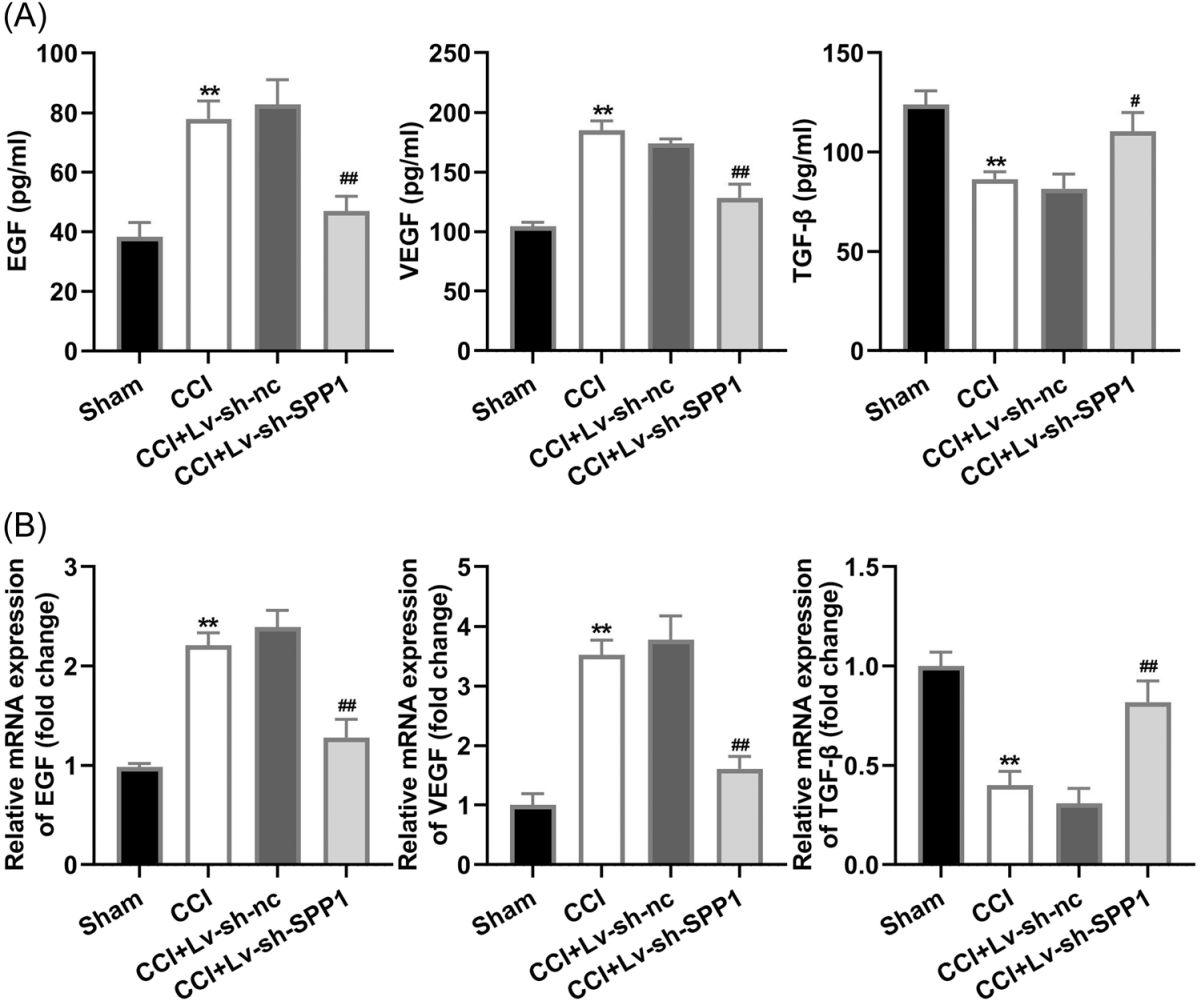
Loss of secreted phosphoprotein 1 (SPP1) inhibits angiogenesis induced by chronic constriction injury (CCI). The levels of epidermal growth factor (EGF), vascular endothelial growth factor (VEGF), and transforming growth factor (TGF)‐β were determined via (A) enzyme‐linked immunosorbent assay and (B) quantitative polymerase chain reaction. **p < .01; ^##^ *p* < .01.

Angiogenesis is evident in inflammation of neurons. Hence, EGF, VEGF, and TGF‐β levels were detected using qPCR and ELISA in dorsal root ganglion from rats. As illustrated in Figure 4A,B, CCI significantly increased the EGF and VEGF levels, while decreasing the TGF‐β levels, and knockdown of SPP1 rescued these CCI‐induced effects. The results indicated that the lack of SPP1 inhibits angiogenesis induced by CCI procedure.

### 3.5 Knockdown SPP1 inactivated the ERK pathway in CCI‐induced rats

**Figure 5:**
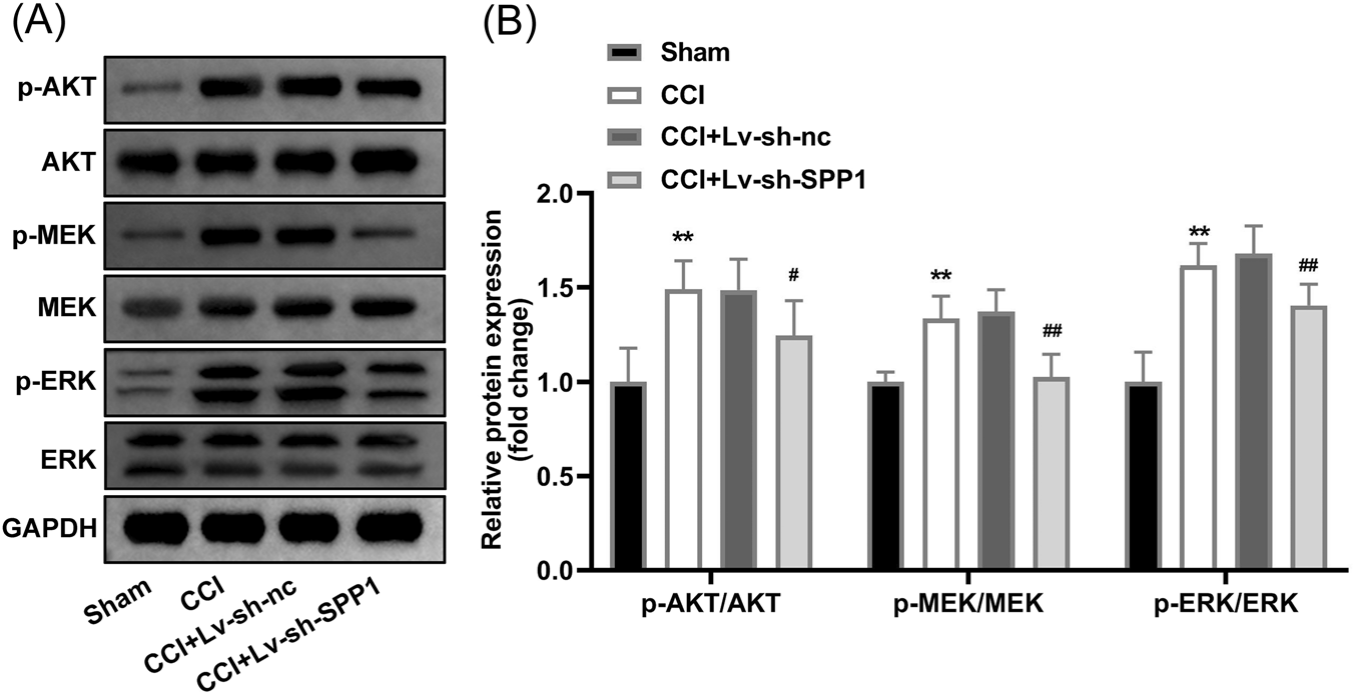
Knockdown secreted phosphoprotein 1 (SPP1) inactivates the extracellular signal‐regulated kinase (ERK) pathway. (A) Serine/threonine kinase (AKT), mitogen‐activated protein kinase (MEK), ERK, p‐AKT, p‐MEK, and p‐ERK protein levels were determined via western blot. (B) The ratios of p‐ERK/ERK, p‐AKT/AKT, and p‐MEK/MEK were quantified. **p < .01; ^##^ *p* < .01.

The effects of SPP1 on the ERK pathway were further explored. CCI surgery significantly increased the protein levels of p‐AKT, p‐MEK, and p‐ERK, while knockdown SPP1 significantly abrogated this effect. However, neither CCI nor SPP1 affected the AKT, MEK, and ERK levels (Figure 5A,B). To sum up, CCI actives the ERK pathway, and SPP1 knockdown reversed this activation.

### 3.6 Silencing of SPP1 relieves NP in CCI‐induced rats via regulating the ERK pathway

**Figure 6:**
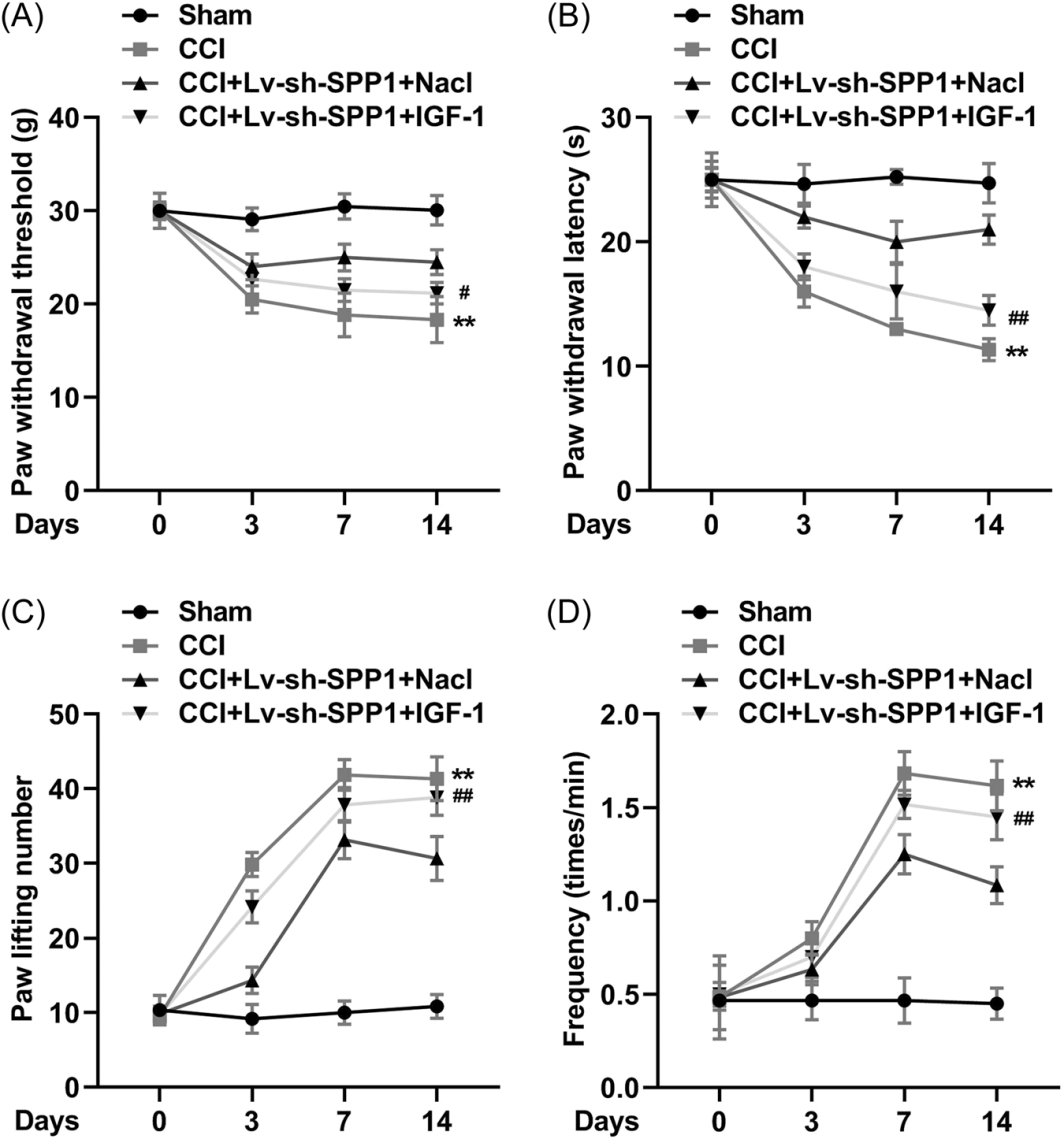
Silencing of secreted phosphoprotein 1 (SPP1) relieves neuropathic pain by regulating the extracellular signal‐regulated kinase (ERK) pathway. (A) Paw withdrawal threshold, (B) paw withdrawal latency, (C) paw lifting number, and (D) frequency were measured following SPP1 knockdown and insulin‐like growth factor 1 treatment. **p < .01; ^##^ *p* < .01; ^#^ *p* < .05.

In CCI‐induced rats, knockdown of SPP1 markedly increased the PWT and PWL, whereas IGF‐1 treatment markedly abolished the effect induced by SPP1 loss (Figure 6A,B). In contrast, IGF‐1 significantly reversed the reduction in the number and frequency of paw lifts induced by SPP1 depletion in CCI‐induced rats. The results demonstrated that silencing of SPP1 relieves pain by inactivating the ERK pathway.

### 3.7 Silencing of SPP1 alleviates inflammation via the ERK pathway

**Figure 7:**
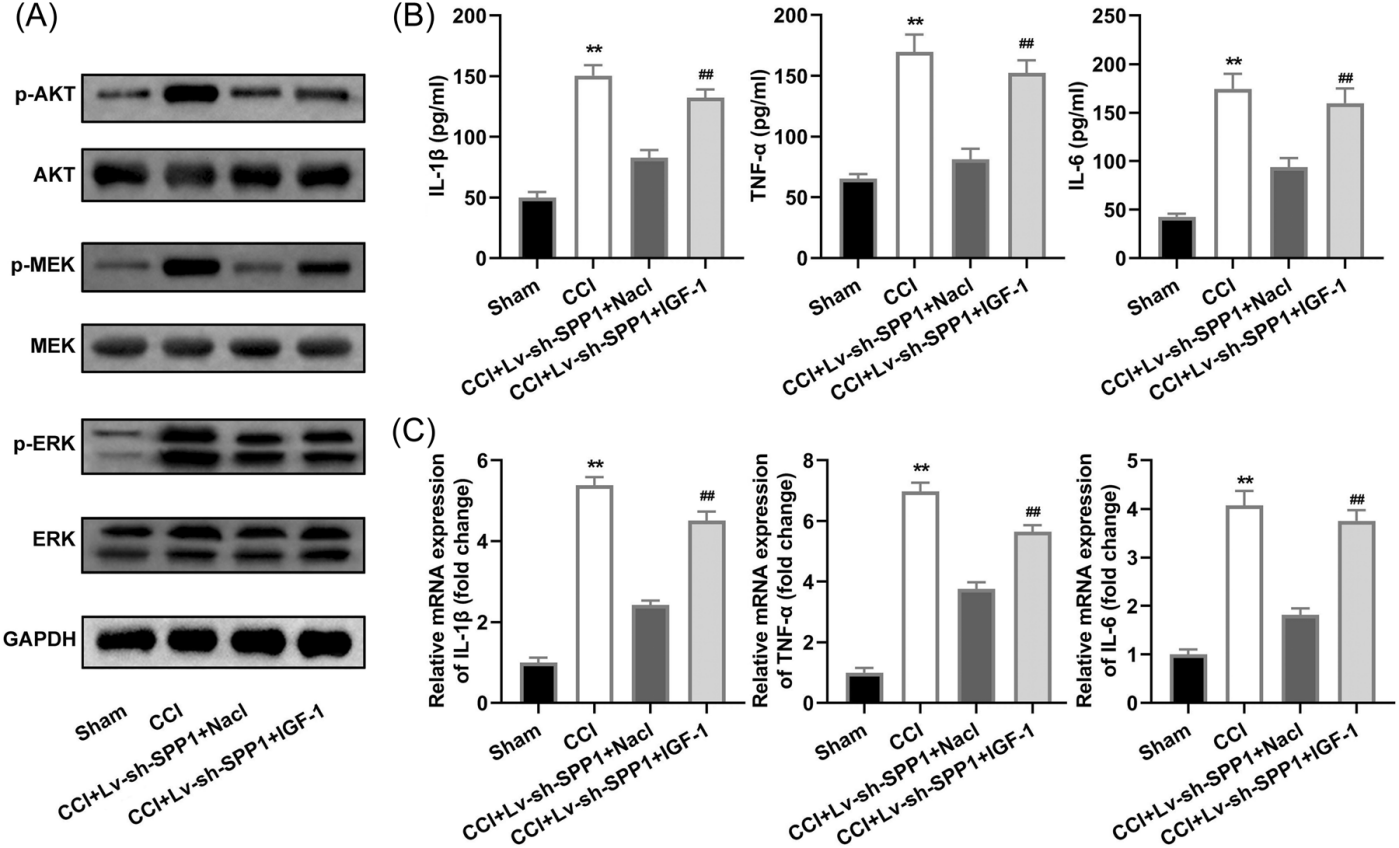
Silencing of secreted phosphoprotein 1 (SPP1) alleviates inflammation via the extracellular signal‐regulated kinase (ERK) pathway. (A) The protein levels of AKT, MEK, ERK, p‐AKT, p‐MEK, and p‐ERK was examined using western blot. The interleukin (IL)‐1β, tumor necrosis factor (TNF)‐α, and IL‐6 levels were tested by (B) enzyme‐linked immunosorbent assay and (C) quantitative polymerase chain reaction. **p < .01; ^##^ *p* < .01.

The protein levels of p‐AKT, p‐MEK, and p‐ERK were downregulated by the knockdown of SPP1, which were significantly elevated by IGF‐1 (Figure 7A) Moreover, the expression levels of IL‐1β, TNF‐α, and IL‐6 reduced by SPP1 knockdown were rescued by IGF‐1 treatment (Figure 7B,C). In summary, activation of the ERK pathway reversed the anti‐inflammation effects caused by SPP1 knockdown in CCI rats.

### 3.8 SPP1 knockdown mediated the ERK pathway to inhibit angiogenesis

**Figure 8:**
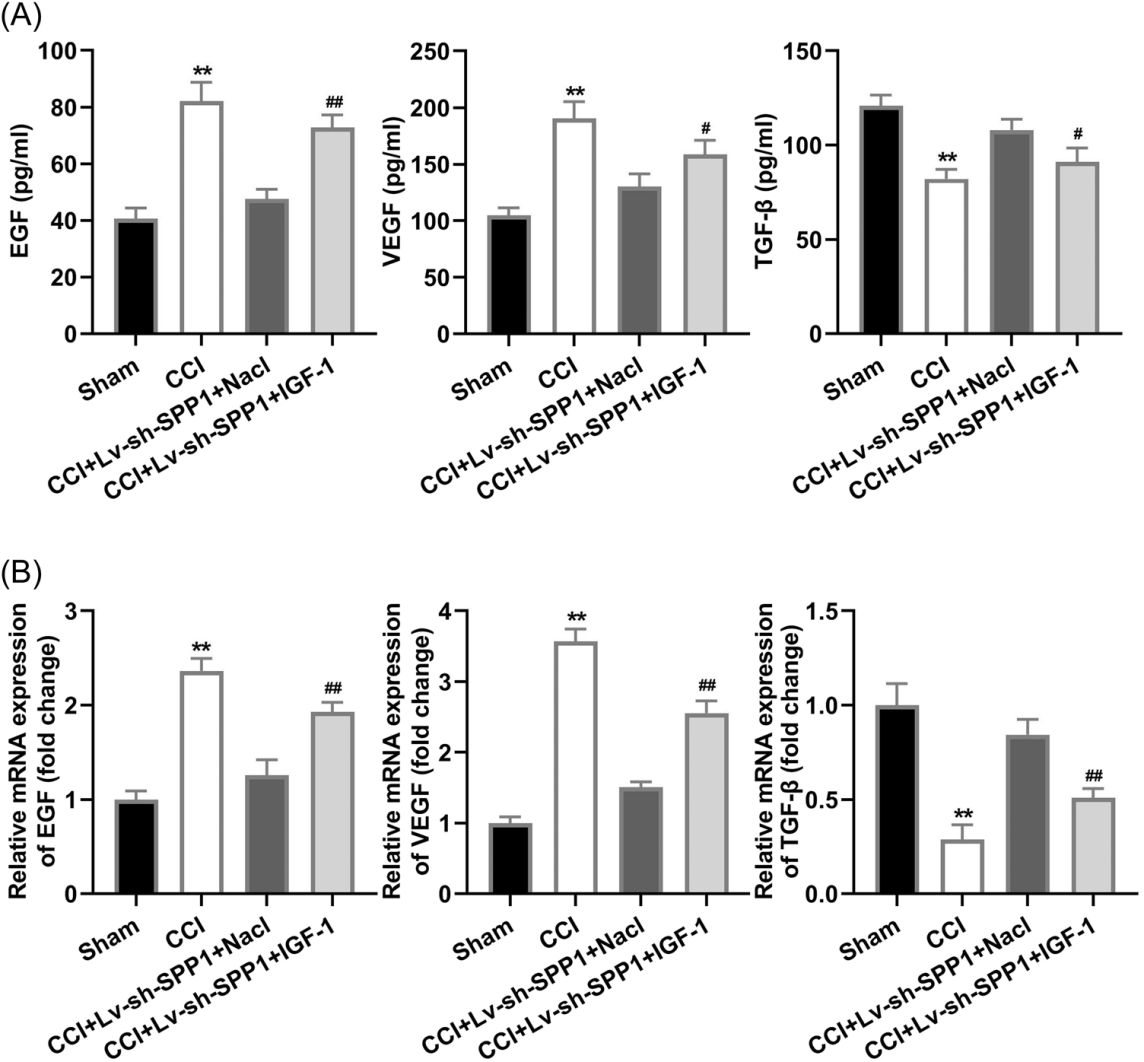
Loss of secreted phosphoprotein 1 (SPP1) suppresses angiogenesis via the extracellular signal‐regulated kinase (ERK) pathway. (A) Enzyme‐linked immunosorbent assay and (B) quantitative polymerase chain reaction were conducted to determine the epidermal growth factor (EGF), vascular endothelial growth factor (VEGF), and transforming growth factor (TGF)‐β levels. **p < .01; ^##^ *p* < .01; ^#^ *p* < .05.

Finally, the results of qPCR and ELISA showed that knockdown of SPP1 decreased the EGF and VEGF levels, but upregulated the TGF‐β levels induced by CCI (Figure 8A,B). These findings suggested that SPP1 knockdown mediated the ERK pathway to inhibit angiogenesis.

## 4 DISCUSSION

Herein, we clarified the role of SPP1 in NP and explored the underlying mechanisms. We established CCI rat model and intrathecal injected shRNA lentivirus to interference with SPP1 expression. Pain behaviors were measured after model establishment. Moreover, inflammatory factors, angiogenesis‐related factors, and ERK pathway were evaluated in the dorsal root ganglion. We found that silencing of SPP1 inhibits inflammation and angiogenesis in CCI rats by regulating the ERK pathway, thereby alleviating NP.

Nerve injury, involving the central and peripheral nerves, is the main cause of NP. Unfortunately, current drugs and technologies are not completely effective for treating NP. CCI is commonly used in model of sciatic nerve injury to explore the pathogenesis of NP. Previous studies have reported that SPP1 is involved in the progression of multiple types of pain.^14^, ^15^, ^16^ Moreover, SPP1 is associated with nerve injury.^30^, ^31^ SPP1 is a key regulatory factor that affects nerve degeneration and regeneration after sciatic nerve injury. However, the role of SPP1 in sciatic nerve ligation‐induced pain remains unclear. Herein, we found that SPP1 is highly expressed in CCI rats, suggesting that it may regulate NP pathogenesis. Moreover, pain intensity, heat sensitivity, and cold sensitivity were evaluated by PWT, PWL, paw lifting number, and frequency. The results in this study indicated that silencing of SPP1 increased PWT and PWL, and reduced paw lifting number and frequency, suggesting that low expression of SPP1 can reduce NP. A previous study has reported that SPP1 is highly expressed in disease‐associated microglia and reflect neuroinflammation.^32^ Indeed, SPP1 expression is increased in spinal cord microglia after injury.^33^ However, this study has not yet explored the localization of SPP1 in the spinal cord. Whether SPP1 is upregulated in spinal microglia after CCI and regulates NP progression is a question that needs to be studied in our future work.

Subsequently, how SPP1 affects NP was explored. Excessive inflammation contributes to the onset and maintenance of NP. Pro‐ and anti‐ inflammatory factors significantly influence NP.^34^, ^35^ The release of pro‐inflammatory factors, including IL‐1β, TNF‐α, and IL‐6, has been widely studied. They are highly expressed in NP, and downregulation of them improves pain to a certain extent.^18^ In addition, IL‐10, an anti‐inflammation factor, is also an antinociceptive cytokine that protects against NP.^36^ Hence, we detected the levels IL‐1β, TNF‐α, IL‐6, and IL‐10 were detected in the dorsal root ganglion of CCI rats. We found that SPP1 knockdown reversed the increase of IL‐1β, TNF‐α, and IL‐6 caused by CCI, rather than affected IL‐10 expression. The results suggested that silencing of SPP1 alleviates the inflammatory environment mainly by inhibiting the release of pro‐inflammatory factors, thus attenuating NP.

Spinal angiogenesis is linked to NP, which is induced by VEGF and plays a crucial role in inflammation.^37^, ^38^ EGF contributes to a number of chronic pain processes by binding to its receptor, EGFR^39^ VEGF, EGF, and TGF‐β are important regulatory factors in angiogenesis.^40^, ^41^ SPP1 has been reported to regulate angiogenesis in multiple diseases.^42^, ^43^ However, whether SPP1 affect angiogenesis in NP remains unclear. In this study, the results showed that silencing of SPP1 reduced EGF and VEGF levels, and increased TGF‐β levels in the dorsal root ganglion of CCI rats, suggesting that silencing of SPP1 relieved angiogenesis in CCI‐induced rats. The findings demonstrated that SPP1 knockdown attenuated the progression of NP by inhibiting inflammation and angiogenesis.

The ERK pathway plays a crucial role in many types of pain and maybe a therapeutic target for NP.^24^ Inhibition of ERK phosphorylation alleviates pain. Activation of ERK signaling activates downstream factors, phosphatidylinositol 3‐kinase, which induces an increase in AKT phosphorylation and regulates cellular processes. There are studies showed that SPP1 is associated with the activation of the ERK pathway.^44^, ^45^ A previous study showed that the changes in SPP1 expression led to alterations in ERK signaling after sciatic nerve injury.^17^ However, this work was mostly at the cellular level, while our study focused on the role of the SPP1–ERK pathway in vivo. In this study, we found that the ERK pathway was activated in CCI‐induced rats, which is consistent with previous studies.^46^, ^47^ Silencing of SPP1 inactivated the ERK pathway. Moreover, IGF‐1 treatment abrogated the effects on pain behavior, inflammation, and angiogenesis induced by SPP1 depletion. Taken together, SPP1 silencing alleviates NP by positively regulating the ERK pathway.

In conclusion, our results demonstrate that SPP1 levels are upregulated in CCI rats, which positively regulate the ERK pathway. Moreover, SPP1 knockdown attenuates the pain behaviors, inflammation, and angiogenesis induced by CCI by downregulating the ERK pathway. Hence, this study suggests the potential use of SPP1 as a target for NP management.

## AUTHOR CONTRIBUTIONS

**Haiyu Xie**: Conceptualization (lead); writing—original draft (lead); formal analysis (lead); writing—review and editing (equal). **Feng Lu and Xiaoling Li**: Software (lead); writing—review and editing (equal). **Enfu Wang and Jiao Mo**: Methodology (lead); writing—review and editing (equal). **Weidong Liang**: Conceptualization (supporting); writing—original draft (supporting); writing—review and editing (equal).

## CONFLICT OF INTEREST STATEMENT

The authors declare no conflict of interest.

## ETHICS STATEMENT

All animal experiments were approved by the Ethics Committee of The First Affiliated Hospital of Gannan Medical University. All procedures were performed in line with the Guide for the Care and Use of Laboratory Animals.

## Supporting information

Supplementary Figure 1.

## Data Availability

The data sets used and analyzed during the current study are available from the corresponding author on reasonable request.
