# Supplementary figures and images for: Silencing of secreted phosphoprotein 1 attenuates sciatic nerve injury‐induced neuropathic pain: Regulating extracellular signal‐regulated kinase and neuroinflammatory signaling pathways
Source: Immun Inflamm Dis. 2024 Feb 2;12(2):e1132. doi: 10.1002/iid3.1132 (PMC10836034; doi:10.1002/iid3.1132)

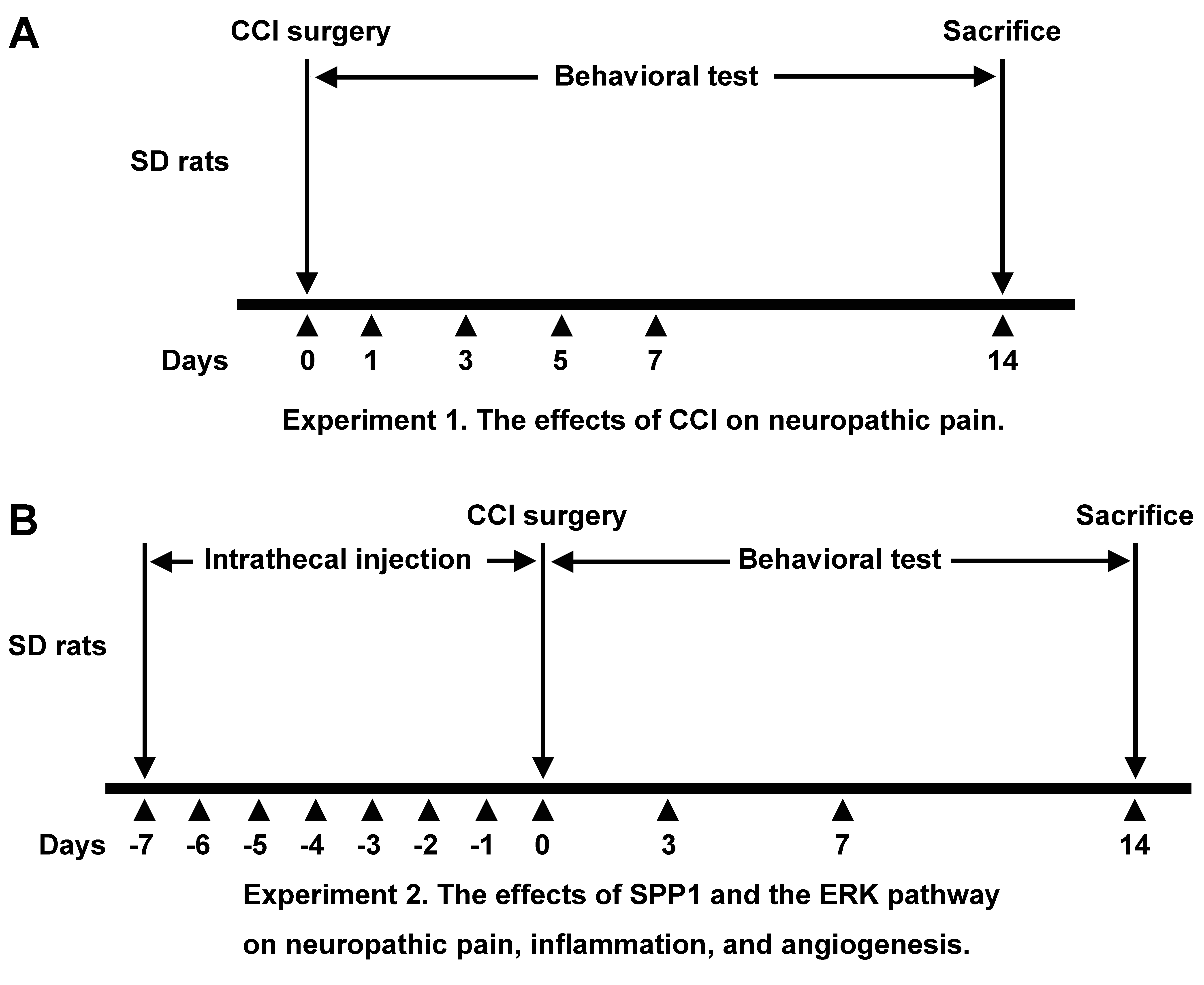

Supplement: Supplementary file 1 — Supplementary Figure 1. [file IID3-12-e1132-s001.jpg]
